# Adipositas und Typ-2-Diabetes (Update 2023)

**DOI:** 10.1007/s00508-023-02184-6

**Published:** 2023-04-20

**Authors:** Martin Clodi, Hermann Toplak, Michael Resl, Johanna Brix, Deborah Raphaela Leitner, Jürgen Harreiter, Friedrich Hoppichler, Thomas C. Wascher, Karin Schindler, Bernhard Ludvik

**Affiliations:** 1 Abteilung für Innere Medizin, Konventhospital der Barmherzigen Brüder Linz, 4021 Linz, Österreich; 2 2. Klinisches Forschungsinstitut für kardiometabolische Erkrankungen, Johannes Kepler Universität Linz, Altenberger Straße 69, 4040 Linz, Österreich; 3 Klinische Abteilung für Endokrinologie und Diabetologie, Universitätsklinik für Innere Medizin, Medizinische Universität Graz, Graz, Österreich; 4 Klinische Abteilung für Endokrinologie und Diabetologie, Universitätsklinik für Innere Medizin, Medizinische Universität Graz, Graz, Österreich; 5 Gender Medicine Unit, Klinische Abteilung für Endokrinologie und Stoffwechsel, Universitätsklinik für Innere Medizin III, Medizinische Universität Wien, Wien, Österreich; 6 Abteilung für Innere Medizin, Krankenhaus der Barmherzigen Brüder Salzburg, Salzburg, Österreich; 7 1. Medizinische Abteilung, Hanuschkrankenhaus, Wien, Österreich; 8 Universitätsklinik für Innere Medizin III, Medizinische Universität Wien, Wien, Österreich; 9 Medizinische Abteilung mit Diabetologie, Endokrinologie und Nephrologie, Krankenanstalt Rudolfstiftung, Wien, Österreich

**Keywords:** Adipositas, Typ-2-Diabetes, Körperzusammensetzung, Ernährung, Formula-Diäten, Obesity, Type 2 diabetes, Body composition, Nutrition, Formula diets

## Abstract

Der Body-Mass-Index (BMI) ist individuell betrachtet ein sehr grobes Maß für den Anteil des Körperfetts am Körpergewicht. Sogar Normalgewichtige können bei Muskelmangel zu viel Körperfett aufweisen (Sarkopenie), weswegen zusätzlich Messungen der Körperzusammensetzung (z. B. Bioimpedanzanalyse [BIA]) empfohlen werden. Lebensstilmanagement mit Ernährungsumstellung und Bewegung ist eine der wichtigsten Maßnahmen in der Diabetesprävention und -therapie. In der Therapie des Typ-2-Diabetes hat das Gewicht als sekundärer Zielparameter zunehmende Bedeutung erlangt. Auch die Wahl der antidiabetischen Therapie, aber auch der Begleittherapien nimmt immer mehr darauf Rücksicht. Die modernen GLP‑1 Analoga als auch der kombinierte GLP-1–GIP-Agonist Tirzepatid nehmen einen wichtigen Stellenwert in der gemeinsamen Behandlung von Adipositas und Diabetes mellitus Typ 2 ein. Die bariatrische Chirurgie ist derzeit bei an Diabetes mellitus Typ 2 erkrankten Menschen mit BMI > 35 kg/m^2^ indiziert und kann zumindest teilweise zur Diabetesremission beitragen, sie muss aber in ein entsprechendes lebenslanges Betreuungskonzept eingebunden sein.

## Abstract (en)

The body mass index (BMI) is a very crude measure of body fatness in individuals. Even normal weight persons can have too much body fat in cases of a lack of muscle mass (sarcopenia), which is why additional measurements of waist circumference and body fatness, e.g. bioimpedance analysis (BIA), are recommended. Lifestyle management including nutrition modification and increase in physical activity are important measures for the prevention and treatment of diabetes. Regarding the treatment of type 2 diabetes, body weight is increasingly used as a secondary target parameter. The choice of anti-diabetic treatment and additional concomitant therapies is increasingly influenced by body weight. The importance of modern GLP‑1 agonists and dual GLP‑1 GIP agonists increases since these drugs target obesity and type 2 diabetes. Bariatric surgery is at present indicated with a BMI > 35 kg/m^2^ with concomitant risk factors, such as diabetes and can lead at least to partial diabetes remission but has to be incorporated into an appropriate lifelong care concept.

Die Adipositas ist auf Basis einer entsprechenden Genetik der wohl wichtigste Risikofaktor für den Typ-2-Diabetes. In der EU sind 17 % der Erwachsenen zwischen 20 und 74 Jahren adipös, 36 % sind übergewichtig [1]. Erhöhter BMI bedeutet erhöhte Mortalität bei Männern und Frauen, wobei dies allerdings auch für Untergewicht gilt („J shaped curve“) [2]. Weltweit ist der größte Teil des Typ-2-Diabetes der Adipositas zuzuordnen, so auch in Europa [3].

Diabetes und Adipositas zusammen erhöhen das Mortalitätsrisiko auf das 7‑Fache [4].

Laut ICD-11 handelt es sich bei Adipositas um eine Erkrankung. Die European Association for the Study of Obesity (EASO) hat sie zum „Gateway of ill Health“, also zu einem zentralen krankheitsbestimmenden Faktor erklärt [5]. Die „Milan Declaration 2015“ der EASO hat die Adipositas als „progressive Erkrankung“ genannt und als zentrales „Tor“ zu vielen anderen Erkrankungen wie die meisten NCDs („non communicable diseases“; nicht übertragbare Erkrankungen) erklärt. So wurde die zentrale Rolle der Adipositas bei Diabetes, Hyperlipidämie und Hypertonie mit der Konsequenz erhöhter kardiovaskulärer Morbidität und Mortalität anerkannt (http://www.easo.org).

Die WHO hat die Adipositas zum größten globalen chronischen Gesundheitsproblem erklärt, das neuerdings die Bedeutung der Malnutrition bei Weitem übertrifft. Im Jahr 2030 könnten neueren Projektionen zufolge etwa 60 % der Weltbevölkerung übergewichtig oder adipös sein [6–8].

Neben den an anderer Stelle behandelten Themen soll der Typ-2-Diabetes im Folgenden aus diesem Blickwinkel betrachtet werden.

## Anthropometrie

Es ist heute erwiesen, dass jeder BMI mit einem unterschiedlichen Körperfettanteil verbunden sein kann. Zwar weisen fast 100 % der Personen mit einem BMI > 30 kg/m^2^ einen hohen Fettanteil auf, aber auch immerhin noch etwa ein Drittel der Normalgewichtigen [9]. Dies ist auf den häufigen Verlust der Muskelmasse und der Muskelkraft mit zunehmendem Alter oder Mangel an Muskelmasse (Sarkopenie) zurückzuführen, die eine ungünstige Fett-Muskel-Relation auch bei Normal- oder Übergewicht bedeutet [10–12]. Viele der genannten Personen werden auch über den erhöhten Bauchumfang entdeckt. Besteht eine abdominelle Fettansammlung, ist das Risiko für Atherosklerose und vorzeitige Mortalität selbst bei normalem BMI erhöht. Als Grenzwerte für den Bauchumfang gelten für eine kaukasische Bevölkerung 88 cm für Frauen und 102 cm für Männer, wobei diese Werte jedoch bei älteren Personen (geringere Körpergröße, Kyphose, Skoliose) nicht anwendbar sind. Da andere ethnische Populationen (z. B. Asiaten) bei gleichem BMI eine größere Fettmasse aufweisen, werden für diese Kollektive andere Grenzwerte diskutiert [13].

Eine korrekte Untersuchung umfasst daher zusätzlich den Bauchumfang und eine geeignete Methode zur Erfassung des Körperfettes („dual energy X‑ray absorptiometry“ [DEXA], BODPOD—„air displacement plethysmography“). Zwar haben Letztere auch ihre Schwächen, geben aber eine gute Orientierung und sind besonders im Verlauf eines Gewichtsmanagements von unschätzbarem Wert [14, 15]. Ein Screening auf Sarkopenie mittels eines Fragebogens SARC‑F kann helfen, eine Sarkopenie frühzeitig zu erkennen [16, 17].

## Implikationen für das Lebensstilmanagement

Beim Gewichtmanagement bevorzugen in den USA und auch weltweit in allen Kulturen Frauen Ernährungsmaßnahmen und Männer Bewegungsmaßnahmen [18, 19]. Eine Zunahme der Muskelmasse und eine Verminderung der Körperfettmasse braucht in der Regel aber bei allen Menschen beides.

Die Basis jeden Lebensstilmanagements liegt in der Einleitung der Steigerung körperlicher Aktivität. Aerobe Bewegung ist zur Verminderung des Körperfettes geeignet [20]. Bei Sarkopenie ist auf eiweißreiche Ernährung in Kombination mit Muskelaufbau durch unterstützendes Krafttraining zu achten [21]. Einen besonders wichtigen Faktor stellt neben der Muskelmasse die Funktionalität dar, weswegen im Monitoring geeignete Parameter empfohlen werden (z. B. „Handgrip-Test“) [22]. Insbesondere im Alter ist die körperliche Fitness von großer prognostischer Bedeutung [23, 24].

Eine erfolgreiche Gewichtsreduktion kann nur mit einer energiereduzierten Diät, die fettreduziert, aber auch kohlenhydratreduziert sein kann und am besten einem mediterranen Ernährungsmuster entspricht, erreicht werden [25–27]. Eine mediterrane Ernährung konnte darüber hinaus bei Menschen mit Diabetes die Notwendigkeit der Verordnung oraler Antidiabetika bei Neudiagnose reduzieren [28].

Im Fall einer fettreduzierten Diät muss die Kohlenhydratqualität beachtet werden (bevorzugt komplexe Kohlenhydrate, möglichst wenig Mono- und Disaccharide).

Wie von Dansinger et al. [29] sehr gut gezeigt, geht es in der Praxis darum, die Patient:innen zu motivieren, ihre Ernährung unter Berücksichtigung der persönlichen Präferenzen zu verändern, die Energiezufuhr zu reduzieren und diese Umstellung dauerhaft beizubehalten.

In der Ernährungstherapie sind heute individuell maßgeschneiderte Kostformen, die auch persönliche Präferenzen, Abneigungen, den kulturellen und religiösen Hintergrund sowie die individuelle ökonomische Situation in Betracht ziehen, zu erstellen.

Supplemente mit definiertem Inhalt können im Ersatz einzelner oder mehrerer (meist 2) Mahlzeiten hilfreich sein („low calorie diets“) [30]. Für kurze Zeiträume können bei entsprechender Eignung der Patient:innen auch stark hypokalorische ketogene Kostformen („very low calorie diets [VLCDs]“) eingesetzt werden, die dann meist von „low calorie diets“ mit 1‑ bis 2 -mal tägigem Mahlzeitenersatz (LCDs) über längere Zeit gefolgt werden [31].

## Gewichtszunahme aufgrund von Begleittherapien

Aus Sicht der Adipositas ist auch darauf zu achten, gewichtssteigernde Begleittherapien zu vermeiden. Dies gestaltet sich jedoch meist schwierig, da sehr viele Arzneimittelgruppen ein gewichtssteigerndes Potenzial aufweisen. Zu diesen sind u. a. auch viele traditionelle Antidiabetika zu rechnen. Im Allgemeinen sind heute jene antidiabetischen Therapien zu bevorzugen, die das Gewicht der Patient:innen nicht steigern, sondern – wenn möglich – eine Gewichtsreduktion unterstützen (Metformin, DPP-IV-Inhibitoren, SGLT-Inhibitoren und GLP1-Analoga) [32].

Generell gibt es einige gewichtssteigernde Begleittherapien, auf welche – wenn möglich – verzichtet oder welche durch gewichtsneutrale oder reduzierende Alternativen ersetzt werden sollen. Hierzu zählen u. a. viele Psychopharmaka, welche zugleich auch die wichtigste gewichtssteigernde Begleittherapie per se darstellen. Des Weiteren sind auch Steroidhormone oder Betablocker für eine moderate Gewichtssteigerung bekannt [32]. Unter Letzteren ist zudem eine gehäufte Diabetesmanifestation zu beobachten [33].

## Benefit von Gewichtsverlust in Diabetesprävention und Therapie

Nachdem die Mehrheit der Patient:innen mit Typ-2-Diabetes übergewichtig oder adipös ist, stellt die Gewichtsreduktion eine wichtige therapeutische Maßnahme in der Diabetesprävention dar. Dass diese einen positiven Einfluss auf das Diabetesrisiko ausübt, konnte bereits in mehreren Studien gezeigt werden [34, 35]. So konnte eine Diabetesreduktion von 58 % in der „Finnish Diabetes Prevention Study“ bei Patient:innen mit Prädiabetes durch Lebensstilintervention (Ernährungs- und Bewegungskonzept) erreicht werden [34]. Ähnliche Resultate wurden auch im „Diabetes Prevention Programme“ erzielt. Hier konnte ein moderater Gewichtsverlust mittels Lebensstilintervention die Diabetesmanifestation um 58 % reduzieren und war damit besser als eine Therapie mit Metformin alleine (ohne Lebensstiländerung) [35]. Des Weiteren belegen epidemiologische Daten den Wert einer frühen Gewichtsreduktion bei Typ-2-Diabetes. Jedes Kilo Gewichtsverlust im ersten Jahr nach Manifestation war in einer Studie von Lean et al. mit einem erhöhten „Survival“ von 3 bis 4 Monaten assoziiert, 10 kg Gewichtsverlust mit einer Wiederherstellung der nach der Diabetesmanifestation verminderten Lebenserwartung im Ausmaß von 35 % [36]. Ein geplanter moderater Gewichtsverlust von etwa 10 kg konnte auch in der „Cancer Prevention Study 1“ der American Cancer Society die Mortalität von Menschen mit Diabetes mellitus um etwa 25 % senken [37]. Diese überaus positiven Ergebnisse wurden auch durch die Resultate der „Look AHEAD study“ belegt. Hier wurde der Gewichtsverlust allerdings auch mit Hilfe von einer Bewegungstherapie erzielt. Durch diese Maßnahmen konnte ein moderater Gewichtsverlust von 5–10 % die Fitness, den HbA_1c_-Level und die kardiovaskulären Risikofaktoren verbessern. Zusätzlich dazu konnten eine Reduktion von Diabetes, cholesterin- und blutdrucksenkenden Medikamenten nach 1 Jahr sowie eine Reduktion von Depressionssymptomen und eine Verbesserung der Schlafapnoe beobachtet werden[38–41]. Interessanterweise ist eine Gewichtsreduktion von > 5 % für diese positiven Effekte vonnöten. Auch in der DiRECT – Studie konnte durch eine Lebensstilintervention bei Patient:innen, die eine mittlere Diabetesdauer von 3 Jahren aufwiesen und ein HbA_1c_ von 7,7 % hatten bei 46 % der Teilnehmer der Interventionsgruppe eine Remission des Diabetes mellitus erreicht werden [42].

Eine dauerhafte Lebensstilveränderung fällt vielen Patient:innen schwer, weshalb eine ergänzende medikamentöse Diabetestherapie angeboten werden sollte.

## Implikationen für die Verwendung antiadipöser medikamentöser Therapien

Nach wie vor wird die Lebensstilintervention als primäre Adipositastherapie betrachtet, welche durch die Gabe von Medikamenten in ihrem Erfolg unterstützt werden kann.

In Europa ist der Triglyzerid-Lipase-Hemmer Orlistat seit etwa 20 Jahren verfügbar, wobei eine rezeptpflichtige Formulation (Xenical®, 3 -mal 120 mg) und eine OTC-Formulation am Markt sind (ALLI®, 3 -mal 60 mg). Orlistat soll gleichzeitig mit einer fettarmen Diät eingesetzt werden (die selbst eine Lebensstiltherapie mit Gewichtsreduktion unterstützt) und reduziert die Fettresorption um weitere 30 %, wodurch der Summeneffekt entsteht. Potenzielle Nebenwirkungen insbesondere wenn eine fettreiche Ernährung die Norm ist, sind in erster Linie gastrointestinaler Natur (Fettstühle, Diarrhöen), insbesondere wenn eine fettreiche Ernährung als Basis genutzt wird.

Darüberhinaus gibt es noch weitere medikamentöse Therapieoptionen. Derzeit sind in den USA mehrere, unterschiedliche Medikamente registriert worden, von denen die Fix-Kombination von Naltrexon und Bupropion auch in Europa von der EMA zugelassen ist [43]. Beide Pharmaka sind seit 2018 auch in Österreich zur Verwendung in der Adipositastherapie erhältlich. Damit ist es möglich, Patient:innen mit Typ-2-Diabetes nicht nur hinsichtlich ihrer Gewichtsreduktion zu unterstützen, sondern gleichzeitig auch ihren HbA_1c_-Wert zu verbessern [44, 45]. Durch diese kombinierte Betrachtung von glykämischer Kontrolle und Körpergewicht (bzw. Muskel- und Fettmasse und deren Relation) eröffnen sich somit völlig neue Strategien in der Diabetestherapie.

Die vielversprechenden Daten für Liragluid, Semaglutid und Tirzepatid, welches den ersten, verfügbaren kombinierten GLP-1-GIP-Agonisten darstellt, erhöhen die Relevanz dieser Medikamente zur Unterstützung der Lebensstilintervention bei adipösen Patient:innen deutlich.

### Liraglutid

Im Rahmen des SCALE-Studienprogrammes wurde Liraglutid in unterschiedlichen Patient:innenkollektiven in Kombination mit einer kalorienreduzierten Diät und Bewegung untersucht.

In der SCALE Diabetes Studie (623 Patient:innen mit Diabetes mellitus Typ 2) erreichten die Patienten einen mittleren Gewichtsverlust von 6 % nach 1 Jahr Behandlungsdauer, bei 25 % der Patienten war der Gewichtsverlust sogar 10 % oder größer [46]. Darüber hinaus ist Liraglutid auch bei Kindern im Alter zwischen 12 und 18 Jahren zur Gewichtsabnahme zugelassen[47].

### Semaglutid

Semaglutid, welches einmal wöchentlich verabreicht wird, wurde im STEP-Studienprogramm untersucht.

In der STEP 2 Studie (1210 an Diabetes mellitus Typ 2 erkrankte Patient:innen) erhielten die Patient:innen 1 mg bzw. 2,4 mg Semaglutid 1‑mal wöchentlich zusätzlich zur lebensstilmodifizierenden Therapie. Nach 68 Wochen erreichten die Patient:innen in der 2,4 mg Gruppe einen mittleren Gewichtsverlust von 9,6 % verglichen mit 3,4 % in der Gruppe mit 1 mg. Zwischen den beiden Gruppen zeigte sich kein Unterschied hinsichtlich der HbA_1c_-Senkung [48].

Die STEP Teens Studie wurde mittlerweile ebenfalls publiziert. Bei adipösen Kindern zwischen 12 und 18 Jahren konnte 2,4 mg Semaglutid den BMI um 16,1 % erfolgreich senken [49].

### Tirzepatid

Der duale GLP-1-GIP-Agonist Tirzepatid zeigte im Rahmen des SURPASS–Studienprogrammes sehr positive Resultate.

In der SUPRASS 1 Studie (478 Patient:innen mit Diabetes mellitus Typ 2 ohne medikamentöse Therapie) erhielten die Patient:innen Placebo oder 5 mg, 10 mg oder 15 mg Tirzepatid. Dosisabhängig erreichten zwischen 31 und 52 % der Patient:innen eine Normoglykämie (HbA_1c_ < 5,7 %) verglichen mit 1 % in der Placebogruppe. Der mittlere Gewichtsverlust lag bei 7,0–9,5 kg [50].

In der SURPASS 2 Studie wurden 5 mg, 10 mg und 15 mg Tirzepatid pro Woche mit 1 mg Semaglutid wöchentlich verglichen. Letztlich konnte für 10 mg bzw. 15 mg Tirzepatid (10 und 15 mg) eine klare Überlegenheit im Vergleich zu Semaglutid gezeigt werden [51].

Die SURPASS 5 Studie untersuchte die Effekte von Tirzepatid 5 mg, 10 mg, 15 mg oder Placebo zusätzlich zu einer bereits etablierten Therapie mit Glargin ± Metformin. Im Vergleich zu Placebo bewirkte Tirzepatid eine zusätzliche Reduktion des HbA_1c_ (5 mg 1,24 %; 10 mg 1,53 %, 15 mg 1,47 %). In der Gruppe, welche mit 15 mg behandelt wurde, lag der Gewichtsverlust bei 10,5 kg [52].

Die Daten aus dem SURMOUNT-Studienprogramm, welches zur Evaluierung von Tirzepatid als Medikament zur Therapie der Adipositas durchgeführt wird liegen aktuell noch nicht vor.

## Bariatrische Chirurgie

Bariatrische Operationen sind momentan die effektivste Strategie für eine langfristige Gewichtsreduktion. Generell kann dadurch eine 15- bis 40 %-Reduktion des Ausgangsgewichtes erreicht werden [53], wodurch sich Adipositas-assoziierte Komorbiditäten wie Diabetes verbessern und die Gesamtmortalität reduziert wird [54–56]. Derzeit ist die Indikation für bariatrische Operationen bei Patient:innen mit Adipositas ohne Diabetes bei einem BMI > 40 kg/m^2^ und bei Patient:innen mit Typ-2-Diabetes bei einem BMI > 35 kg/m^2^ gegeben. Aufgrund der positiven Effekte werden bariatrische Operationen in manchen chirurgischen Zentren bereits bei viel niedrigerem BMI durchgeführt. Hierfür sind nach wie vor sorgfältige Nutzen-Risiko-Analysen aus kontrollierten Studien abzuwarten. Auch wenn dadurch die bariatrische Chirurgie einer immer größeren Patient:innenpopulation zugänglich gemacht wird, sollte die Patient:innenwahl nach wie vor äußerst gewissenhaft erfolgen, um jenen Menschen die erforderlichen Basis- und Kontrolluntersuchungen vor und nach bariatrischen Operationen zu ermöglichen. Neben der Suche nach Mangelerscheinungen sollten auf jeden Fall die Körperzusammensetzung (Muskel-Fett-Relation) und auch die Knochendichte – erstmals spätestens 2 Jahre nach der Operation – untersucht werden [57]. Da die multidisziplinäre medizinische Nachbetreuung dieser Patient:innen sehr umfangreich ist, wurden dazu 2 Übersichtsarbeiten zu den Richtlinien publiziert [58, 59].

In letzter Zeit wurde die bariatrische Chirurgie auch immer wieder als mögliche Therapieoption für Typ-2-Diabetes betrachtet, da sie sowohl zu einer signifikanten Verbesserung der Hyperglykämie beitragen als auch zu einer kompletten Diabetesremission führen kann [53, 60]. Daher wird sie in diesem Zusammenhang auch immer öfter als „metabolische Chirurgie“ bezeichnet und als Behandlungsmöglichkeit für Typ-2-Diabetes bei ausgeprägter Adipositas empfohlen [61, 62]. Generell beträgt die Diabetesremissionsrate 45–95 %, je nachdem, welche Operation durchgeführt wurde – wobei ein größerer Gewichtsverlust zu einer höheren Diabetesremission beiträgt [63–65]. Im direkten Vergleich konnte gezeigt werden, dass bariatrische Operationen mit antidiabetischer Therapie einer rein medikamentösen Therapie hinsichtlich der Gewichtsreduktion, der glykämischen Kontrolle und dem Gebrauch von Blutzucker-regulierenden, Blutdruck-senkenden und Cholesterin-senkenden Medikamenten überlegen sind. Die Daten des erst kürzlich erschienen 5‑Jahres-Follow-up-Berichtes von „STAMPEDE“ (Surgical Treatment and Medications Potentially Eradicate Diabetes Efficiently) konnten zudem zeigen, dass die positiven Effekte auch noch 5 Jahre nach der Operation anhielten. Dennoch sollten bariatrische Operationen in ihrem Erfolg nicht überschätzt werden („Heilung des Diabetes“), da auch sie eine neuerliche Manifestation des Diabetes oder eine Verschlechterung desselben nach Jahren nicht ausschließen können [53]. Daher und auch wegen der möglichen Spätfolgen der Operationen sind weitere Langzeitstudien vonnöten, welche sich mit diesen Fragestellungen auseinandersetzen.

## Interessenkonflikt

M. Clodi hat von folgenden Unternehmen, die auch fördernde Mitglieder der ÖDG sind, Forschungsunterstützungen und/oder Honorare erhalten: Abbott Diabetes Care, AstraZeneca, Boehringer Ingelheim, Daiichi-Sankyo, Dexcom, Eli Lilly, Medtronic, MSD, Novartis, Novo Nordisk, Sanofi. H. Toplak hat von folgenden Unternehmen, Forschungsunterstützungen und/oder Honorare erhalten: Almased, Bodymed, Cheplapharm, Eli Lilly, Insumed, MSD, Mylan, Novartis, Novo Nordisk, Pfizer, Sanofi-Aventis, Servier, Omron, Deutschland, Takeda, Vivus. M. Resl hat von folgenden Unternehmen, die teils auch fördernde Mitglieder der ÖDG sind, Honorare für Vorträge und/oder Beratertätigkeit bzw. Fortbildungsunterstützung erhalten Boehringer Ingelheim, Mylan, Dexcom, Abbott J. Brix: Honorare für Advisory Boards und Vorträge von Astra Zeneca, Boehringer-Ingelheim, Eli Lilly, Novo Nordisk, Sanofi. D.R. Leitner hat von folgenden Unternehmen Forschungsunterstützungen und/oder Honorare erhalten: Novo Nordisk. J. Harreiter hat Forschungsförderung und Vortragshonorare bzw. Fortbildungsförderung (Kongressunterstützung) von den Firmen Astra Zeneca, Novo Nordisk und Böhringer Ingelheim erhalten. F. Hoppichler hat Forschungsförderung und Vortragshonorer von folgenden Firmen erhalten: Boehringer Ingelheim RCV GmbH & Co KG, Sanofi-Aventis GmbH, Daiichi Sankyo Austria GmbH, AstraZeneca Österreich GmbH, Amgen GmbH, Novo Nordisk Pharma GmbH, Eli Lilly Gesellschaft Gm.b.H. T.C. Wascher hat Forschungsunterstützung, Honorare für Beratung oder Fortbildung erhalten von Boehringer Ingelheim, Astra Zeneca, Ely Lilly, Novo Nordisk. K. Schindler gibt an, dass kein Interessenkonflikt besteht. B. Ludvik erhielt Honorare für Vorträge, die Teilnahme an Advisory Boards und Forschungsunterstützung von: Amgen, Astra Zeneca, Bayer, Boehringer Ingelheim, Daiichi Sankyo, Eli Lilly, MSD, Novartis, Novo Nordisk, Sanofi.
